# Digital and Financial Literacy for Uplifting Women and Achieving Sustainable Development Goals

**DOI:** 10.12688/f1000research.156744.1

**Published:** 2024-11-14

**Authors:** Deepak Mishra, Vinay Kandpal, Naveen Agarwal, Rakesh Kumar, Rajesh singh, Neeraj Priyadarshi, Bhekisipho Twala

**Affiliations:** 1 School of Business, University of Petroleum and Energy Studies, Dehradun, Uttarakhand, 248007, India; 2 Department of Management Studies, Graphic Era Deemed to be University, Dehradun, Uttarakhand, 248002, India; 3 Centre for Continuing Education (UPESON), University of Petroleum and Energy Studies, Dehradun, Uttarakhand, 248007, India; 4 Uttaranchal Institute of Management, Uttaranchal University, Dehradun, Uttarakhand, India; 5 Uttaranchal Institute of Management, Uttaranchal University, Dehradun, Uttarakhand, India; 6 Department of Electrical Engineering, JIS College of Engineering, Kalyani, West Bengal, India; 7 Digital Transformation Portfolio, Tshwane University of Technology, Pretoria, Gauteng, South Africa

**Keywords:** Digital literacy, financial literacy, Women's empowerment, Sustainable development goals, Economic independence, Entrepreneurial amenities, Socioeconomic barriers

## Abstract

Digital and financial literacy are changing the landscape of the globe in terms of its approach to development. These two literacies also help to achieve women’s empowerment and, consequently, the sustainable development goals (SDGs) more quickly. This systematic literature review looks at the two literacies and their effects, focusing on women’s participation and showing how digital literacy not only increases women’s access to knowledge and connectivity but is also a door to women’s entrepreneurship and financial independence. Likewise, financial literacy, when coupled with digital skills, can be a breakthrough to the traditional socioeconomic barrier – poverty – by assisting women in making informed economic decisions, increasing their financial independence and resilience in the face of a global crisis. Empirical realities that still hinder women’s education, such as cultural norms, infrastructural deficiencies and educational gaps, still exist. However, the paper points out that to have a more equal world based on the SDGs, the double literacy strategy for women’s empowerment should not be negotiable.

## 1. Introduction

The modern digital world is quite different from the world we would have known only a few decades ago. Significant developments in technology and digital transformation have already transformed several industries and aspects of our lives in significant ways. Globally, the factors that reflect a country’s development and progress includes financial inclusion, literacy, and female workforce participation ( Duflo, 2012). Nonetheless, this also involves women in household financial decisions, which results in better financial health ( Haque & Zulfiqar, 2016).

There is already evidence that greater female financial independence leads to more spending on families, resulting in broad societal benefits such as greater health and education ( Doepke et al., 2011). There is also evidence that greater female employment and earnings correlate with better economic growth and lower gender wage gaps ( Goldin, 2014). For women in settings with societal or regulatory barriers, digital platforms currently offer greater opportunities than ever before for earning and learning ( World Bank, 2016) and building cross-regional networks and collaborations to break down the insider silos of the past ( Huyer, 2016). Identifying the cross-cutting power of women’s empowerment (SDG 5) on other SDGs (e.g., reduced poverty, SDG 1; improved health, SDG 3; and others) is critical ( UNDP, 2016). In addition, the positive connections between financial and digital literacy and SDGs such as Quality Education (SDG 4) and Decent Work (SDG 8) are emerging ( UNESCO, 2017), and more detailed insights into the nexus of literacy, women’s empowerment, and SDGs can facilitate more focused, targeted, and effective policy interventions ( World Bank, 2018).

Financial literacy positively relates to intelligence, where meta-analyses report moderate to large effect sizes. Studies show that financial literacy has become more important over time due to increased responsibility for personal retirement planning, growing access to financial goods, the rise of the gig economy, and innovations in fintech services. Financial literacy has also become more relevant amid the recession caused by the COVID-19 pandemic, as individuals face higher levels of financial distress. A recent analysis using survey data spanning 2015-2020 from the US Federal Reserve Board reveals that Americans’ sentiment about their financial lives had significantly improved prior to COVID-19. However, financial stress showed an overall increase following the pandemic, which also caused unemployment rates to soar ( Morgan et al., 2019; Walker et al., 2023).

In recent years, some scholars have seen growing interest in examining the impact of financial literacy on people’s economic decisions. Simultaneously, there has been an increasing provision for understanding and measuring the concept of financial literacy. Today, financial literacy learning has become an essential self-management skill for people living in a family and business activities ( Vieira et al., 2020). Rapid changes in the global economy require financial literacy to adapt to new financial models ( Demirguc-Kunt et al., 2018). It is seen as a predictor of individual differences in investor attitudes. Cryptocurrencies are negatively associated with higher levels of intelligence after controlling for financial literacy and narcissism, potentially due to the mechanism of cynicism.

The rationale for researching digital and financial literacy in relation to women’s empowerment and the SDGs is based on the premise that the empowerment of women is a condition not only of social equity but also of the innovation and efficacy of approaches aimed at attaining ever more ambitious and transformative development goals. In the 21st century, digital and financial literacy will be the key to ensuring that the individuals and communities of today and tomorrow have the capacity to adapt, innovate and prosper. This review will set out how digital and financial literacy affect women’s empowerment and the extent to which women’s empowerment, in turn, affects the framing and delivery of the SDGs. We will map out the literacies’ direct and indirect impacts, highlight successful global initiatives, highlight current challenges, and develop recommendations that can inform policy and strategy for women’s empowerment through improved literacy.

## 2. Literature review

### 2.1 Financial well-being and decision making

Financial well-being is the state of satisfying all of one’s current and ongoing financial obligations, feeling secure about one’s financial future, and making life-enhancing decisions ( Hudson et al., 2022). More specifically, an individual’s financial well-being corresponds to the extent to which the individual feels that he or she (1) has control over day-to-day and month-to-month finances, (2) can absorb a financial shock, (3) is on track to meet his or her financial goals, and (4) has the financial independence to make the decisions that permit one to appreciate life ( Weida et al., 2020). Although specific goals and visions of a good life vary from person to person, these four elements reflect two common and consistent themes: security and freedom of choice in the present and for the future. Retirees display high financial literacy, and together with debt anxiety, this is significantly correlated with resource allocation preferences. Financial literacy reduces debt anxiety in men, increases women’s risk tolerance, and is associated with a higher priority for KiwiSaver. Greater debt anxiety is associated with debt repayment, and debt avoidance in old age is critical to long-term well-being ( Noviarini et al., 2023).

### 2.2 Financial literacy

Financial literacy refers to the comprehension and awareness of financial principles and potential hazards, along with the abilities, drive, and self-assurance to utilize this knowledge and understanding to make sound choices within diverse financial situations. Its objective is to enhance the financial well-being of individuals and society and promote active involvement in economic activities ( Méndez Prado et al., 2022). As in the definition of any PISA domain, the first part of the definition concerns reasoning and behavioral traits that ‘are characteristic of the domain,’ and the second part focuses on why literacy in that specific domain was defined. We find a positive relationship between people with higher levels of financial literacy and persons inclined to Socially Responsible (SR) financial investments, and people inclined to SR financial investments are more likely to favor SR financial intermediaries. Compelling evidence of our result emerges by using different numbers of neighbors. SR investors or those with a higher level of financial literacy are more likely to favor SR financial intermediaries. This implies that in order to attract financially literate customers or SRI retail investors, financial companies must do more than offer traditional and SRI products. In order to become sustainable intermediaries, they need to adopt environmental, social and governance (ESG) strategies ( Cucinelli & Soana, 2023).

There is a plethora of studies that have examined the link between financial inclusion, financial literacy, and sustainable development and how they are mutually enhanced. However, there is a dearth of literature that explicitly explores this link from the lens of rural agrarian communities, which require development opportunities in finance rather than financial literacy ( Akande et al., 2023), and where a more important emphasis should be placed on the role of financial literacy in influencing and improving financial behavior. This link between financial literacy and financial behavior has important implications. The role of limited attention in explaining the positive association between financial literacy and formal bank account ownership, financial market participation, commercial insurance, pension plan ownership and credit card ownership supports the view that individuals with higher levels of financial literacy are more likely to benefit from such counsel due to the ability of greater financial literacy to improve access to specific financial information ( Xu et al., 2022). Therefore, we reason that ceteris paribus, individuals with higher financial literacy are more likely to engage in the pursuit of such counsel actively. Furthermore, the extant literature reveals that there is a positive correlation between financial literacy and financial behavior, and specifically, those requiring financial counseling with greater levels of financial literacy are more likely to utilize financial counsel ( Liu & Lu, 2023).

### 2.3 Digital financial literacy

The ability to use digital technologies in an effective and responsible manner is referred to as digital literacy. It is inextricably tied to the specific skills, knowledge, and attitudes that lead to utilizing digital devices, applications, and online platforms for communicating, finding information, critiquing, problem-solving, innovating, and creating. The use of Industry 4.0 technologies, such as financial technology (Fintech) and the strategic utilization of organizational resources are major drivers of long-term sustainability and operational efficiency in firms ( Siddik et al., 2023).

Financial literacy refers to its knowledge, skills, and understanding of financial ideas and principles to make informed choices about financial resources. In the concept of financial literacy, we can find several issues connected with personal finance, including budgeting, savings, investment, borrowing, and debt management. Financial literacy links with the knowledge and understanding of available financial products and services, such as bank accounts, loans, credit cards, insurance, and investments. It also refers to the knowledge of financial information, including statements, budgets, and other documents needed to manage finances, and the ability to analyze what the financial information is delivering for the individual. It also concerns smart decision-making about finances to enable the accomplishment of some short- and long-term financial goals ( Lusardi & Mitchell, 2014). Financial capabilities work as a mediator between financial literacy, digital financial literacy, and financial decision-making. DFL is a new concept, and it has received less attention in the field of academic papers. So far, fewer empirical papers have explored the direct effect of DFL on financial decision-making. Skills in the specific domain had a direct impact on decisions made about investing and, consequently, on perceptions of financial well-being. Digital financial literacy, being an indicator of financial decision-making, also emerged as a direct predictor and mediator. The impact of financial aptitude and autonomy as mediators of financial decision-making and well-being is clearer. On the other hand, the influence of impulsivity is almost insignificant in relation to financial decision-making ( Kumar et al., 2023).

### 2.4 Women empowerment

The idea of women’s empowerment is about enabling and increasing women’s social, economic, political, and personal resources and capabilities; the concept includes creating an environment in which women are free to make choices and decisions about their lives and work with equality. The advancement of women’s empowerment yields advantages not only for women themselves but also for the broader societal framework. Women’s empowerment enables them to contribute more effectively to various aspects, such as economic growth, poverty reduction, sustainable development, and social progress. This phenomenon fosters a societal environment characterized by inclusivity and equity when the rights and capabilities of every individual are acknowledged and esteemed. Women’s empowerment refers to the systematic progression through which women attain authority and autonomy, enabling them to exercise deliberate decision-making. It is suggested that there is a need for more targeted policies that can effectively address the existing gender gap to promote comprehensive financial inclusion ( Ghosh, 2022).

The emergence and contribution of women entrepreneurs to the national economy have played a crucial role in the development of a burgeoning nation. Women have commenced making contributions to economic development through their distinct professional endeavors. Women have transitioned from their traditional roles as homemakers to become influential business leaders with the skills and determination to manage a firm and overcome obstacles for their advantage effectively. The increasing IT environment has created extra opportunities for women entrepreneurs to enhance the growth and success of their companies. One crucial aspect that is widely acknowledged as significant in decision-making and organizational activities is the ability to conceptualize and benchmark work. The promotion of positive behavior in women’s entrepreneurship within a specific cultural and societal context serves to enhance empowerment ( Andriamahery & Qamruzzaman, 2022).

### 2.5 Financial resilience

The terms “resilience” and “resiliency” have gained significant prominence in discourse since the onset of the mortgage meltdown and subsequent financial crisis. Financial resilience refers to an individual’s capacity to endure and recover from life circumstances that harm their income or assets. The longer-term perspective pertains to one’s connection with finances. Financial inclusion initiatives aim to improve the accessibility of financial services for vulnerable populations, fostering greater inclusivity and financial resilience within societies. The rise of the fintech industry and digital means of payment, coupled with the fact that more than two-thirds of the world’s population now own a mobile phone, all indicate how digital financial literacy is now taking hold in societies worldwide. In response to this shift, it is time to reimagine financial literacy by blending it with digital literacy – something that has important implications for countries aspiring to adopt either a dual-track or a combination approach to improving the long-run resilience of households ( Kass-Hanna et al., 2022).

India has the highest percentage of inactive bank accounts in the world, at 35 percent ( Dhanamalar et al., 2020). According to the Findex survey, 2021, more than 32 percent of women-owned bank accounts in the country are inactive. The country shows the highest gender gap in inactivity, with a difference of 12 percentage points. Surprisingly, fewer than one in five banked women save formally with the bank ( World Bank, 2021).

## 3. Methods

We present systematic literature review (SLR) methods to provide an overall analysis and constructive evaluation that investigate, identify, and offer rectification for possible issues for future research. According to Massaro et al. (2016), SLR is a systematic approach to analyzing scholarly literature to identify insights, provide critical reflections, offer new research directions, and ask research questions.

The SLR method collects all the articles on a particular research question and examines it in a systematic manner ( Mukherjee et al., 2017). It helps make the review impartial and transparent. It is exhaustive and not as casual as a traditional review ( Bryman, 2012; Choong, 2014). This study first searched, found and reviewed the financial inclusion and sustainable development literature published from 2013 to 2023 using key terms such as ‘digital and financial literacy,’ ‘financial well-being’, ‘financial resilience’, ‘financial decision making’, ‘investment behavior’, ‘women’s empowerment’, and combines them into the subject level. The papers were then filtered after setting the scope of the review by using the scientific qualification type such as ‘empirical, review papers’, etc. Articles were investigated first and sieved from high-quality and high-impact journals, which are listed in electronic databases such as Scopus, Web of Science (WoS) /Social Science Citation Index (SSCI)/Journal Citation Report, which holds the list of academic journals with an IF. This search helps to find possible sources of review ( Paul et al., 2017). Literature search must be extensive and use as much database as possible. It is advisable to use many databases with a risk of getting duplicate articles, but it minimizes the chance of missing a significant publication ( Vieira & Gomes, 2009). The initial stage of the literature search identified 1045 research articles. Further work was done to refine 85 papers, which were studied and scrutinized for the review type and classification of research, and to categorize them by type of study primarily focusing on India and emerging economies. Conference proceedings and book chapters were excluded from the research work. Extractions from Scopus and WOS for this study were made on December 30, 2023. The articles selected were analyzed and understood thoroughly, considering the topic’s relevance. The research also included secondary data sources such as reports published by institutions such as the World Bank, Asian Development Bank, UNESCO, and ILO.

## 4. Discussion

The 2030 Agenda for Sustainable Development advocates for a novel and transformative vision. It outlines 17 linked and indivisible Sustainable Development Goals (SDGs), prioritizing gender equality. Gender equality and women’s empowerment are explicitly prioritized through stand-alone Goal 5 and are integrated throughout the Sustainable Development Goals (SDGs). The UN 2030 Agenda acknowledges multidimensional inequality both inside and within nations, committing to the principle of “leaving no one behind.”

The Asian Development Bank (ADB) and the UN Women’s Regional Office for Asia and the Pacific collaboratively conducted the inaugural extensive assessment of gender equality and women’s empowerment in the Asia and Pacific region within the context of the Sustainable Development Goals framework (The Asian Development Bank & UN Women, 2018). Financial inclusion is regarded as a vital metric of progress and is recognized as a facilitator for at least eight of the 17 Sustainable Development Goals (SDGs). Access to bank accounts, loans, insurance, and other financial services directly enhances health, education, and employment results.

In turn, such progress helps achieve the collective goals of eradicating poverty, promoting inclusive growth, and reducing inequality. Every transformation initiative delineates crucial investments and regulatory obstacles, necessitating coordinated efforts from certain government entities in collaboration with the private sector and civil society. Hence, it is possible to implement transformations inside governmental frameworks while acknowledging the significant interconnections among the 17 Sustainable Development Goals (SDGs) ( Sachs et al., 2019).

### 4.1 Synergy between digital, financial literacy, women’s empowerment and SDGs

Mapping emergent opportunities for digital and financial literacies could be likened to the interlacing of two strands of thread. Each strand both complements and strengthens the other and, depending on how much it is intertwined, the combination yields a stronger, more dynamic whole. The sum of these two literacies together is greater than the two components on their own. The integration of digital and financial literacies can improve favorable outcomes by the following:

**Increased access and outreach**: Digital democratization of financial knowledge has the potential to increase access and outreach by providing members of the public access to a wider range of financial education materials, tools, and services via the Internet. One of the major benefits of the digital age has been the democratization of financial knowledge, which has allowed many people, irrespective of where they live or what their economic status is, to have access to this information via online courses, apps, or websites.

**Better financial decision-making:** The digitally savvy person can use a range of online tools, such as budget trackers, investment apps, and online banking, to help make sound financial decisions. Organizations can quickly assess, evaluate, and act on their financial data, thus making better use of their financial information and, as a result, having better financial governance.

**Empowerment due to Information**: Information is widely available on the Internet, and people can use it to become empowered. Women now have access to information on their rights, benefits, and opportunities via the Internet, which helps them earn more money and be more independent.

**Better economic participation**: Digital technologies enable women to gain better economic participation by taking advantage of the gig economy, e-commerce or other online ventures with digital financial literacy, thereby improving their economic status.

**Security and transparency**: As long as someone has a good understanding of the process involved, digital financial transactions can be more transparent and secure than more traditional methods. In places where women’s access to funds may be restricted, women must be able to exercise covert control over their resources.

**Broader networks and horizons**: Digital literacy means access to networks, communities and markets beyond the geographic area, providing opportunities and potential for growth. With connectivity, alongside financial literacy, greater entrepreneurial opportunities and the possibility of forming mutually productive partnerships could come.

**Personalized digital financial solutions**: Through advanced digital financial platforms enabled by AI and ML technologies, users can receive personalized digital financial recommendations to enhance their financial well-being. Users with sufficient digital literacy skills can explore and access these personalized recommendations to their advantage.

**Promotion of Sustainable Finance**: Although the goals of sustainability are gaining ground at an international level, it is still incumbent upon digital platforms to promote sustainable finance so that people can make the right investment decisions. Digital literacy helps in procuring and utilizing the information and analysis available regarding ethical investments and sustainability-focused financial goals. People with digital literacy can match their financial strategy to the goals of global sustainability.

Women can use green microfinance to increase their financial literacy and become more empowered, thanks to their local knowledge and experience. In this case, the indigenous knowledge came from the women’s adherence to the ancestral worship tradition for their Marapu deities. The study aimed to understand if strengthening the pro-literacy (SDG 4) policy could help the government at the local level achieve its goals related to gender equality (SDG 5) and climate change (SDG 13) ( Pathak et al., 2022).

Digital literacy provides access to a vast digital space with tremendous opportunities, while financial literacy provides the necessary understanding to make the best use of the space. Together, they provide an enabling environment for individuals, especially those who are disadvantaged, such as women, to get access to resources, make informed choices, and build a resilient economic life. The integration process is not only to help people develop their agency but also to promote the growth of the overall economy and stability and further the goal of building a community with a shared future for mankind, a community of shared destiny that is inclusive, fair, and rich. There is a persistent inequality in the access, use, and benefits of digital financial services between genders and socioeconomic statuses and between urban and rural areas in developing countries. By the time we finished the study, we made several recommendations. Most of them were related to better digital infrastructure, simplifying the complex nature of conventional banking, and promoting the importance of financial education, all to support the development of digital financial inclusion in the world ( Tay et al., 2022).

Patriarchy as a structural barrier, psychological barriers, low income/wage, low financial literacy, lack of financial access, and ethnicity are a few of the barriers that were identified as hindering women’s empowerment. Our extensive literature survey further identified six well-evidenced interventions that were brought about by the government and corporate sector, namely: government/corporate programmes and policies, microfinance, formal savings accounts and services, cash and asset transfer programmes, self-help groups, and digital inclusion. To conclude, although ample research has been conducted in this area, still the literature has excessive gaps, which need to be explored further.

### 4.2 Underpinning theories

The present study investigates two crucial theories. The theory of planned behavior (TPB) and the technology acceptance model (TAM) are two prominent theoretical frameworks that have significantly contributed to the comprehension of human behavior, particularly in relation to the acceptance and utilization of technology.

The theory of planned behavior suggests that individuals adopt a very rational decision-making process where they weigh up the pros and cons of their chosen course of action. The theory of reasoned action and the theory of planned behavior both apply to situations where there are arguments for choosing to act or arguments for not choosing to act. According to the theory of planned behavior, the intention is the main determinant of behavior – and what we intend to do is to either do or not do a particular thing. Three aspects of preferences influence the formation of intentions: (i) attitude, that is, one’s evaluation of whether to do or not do a particular thing; (ii) subjective norm or one’s perception of whether other people think that one ought to do, or not do, a particular thing; and (iii) perceived behavioral control or one’s perception of how able or unable one is to do the thing in question.

The Theory of Planned Behaviour, one of the most popular theories in social psychology, has been extended by adding perceived behavioral control to offer a complete account of people’s behavioral intentions. General, specific, and situational goals, acting together with measures of behavioral control beliefs, allow for precise predictions of diverse behavioral intentions. Goals plus behavioral control beliefs can account for the range of actual behavior. Together with attitudes and subjective norms, perceived behavioral controls are associated with distinct sets of salient behavioral, normative, and control beliefs, which are specific to the target behavior under consideration. However, the exact nature of these relations remains to be specified. The theory identifies and draws upon three distinct groups of factors.

1) Perspectives or dispositions
2) Subjective norms and prescriptive influence

Perceptions of behavioral control refer to a person’s belief that he or she can perform a behavior or task adequately, which recognizes the many factors (internal and external) that can affect the person’s capacity for action.

1) **The Impact of Attitudes on Financial Decision Making**:
An attitude is a predisposition, affective orientation, or learned tendency to respond favorably or unfavorably towards an idea, individual, or instance. It constitutes the enduring mental framework an individual employs in their interpretation and evaluation of a concept, entity, person, or situation ( Vargas-Sánchez et al., 2016). There is also evidence suggesting that financial attitude and financial literacy are positively correlated with the choice of financial instrument, and financial behavior serves as a mediating factor. Rai et al., (2019) investigated whether financial attitude can enhance decision-making and whether financial behavior can mediate this process. The results provide a solid basis for their belief that financial attitude and financial literacy have a positive influence on financial behavior.
2) **Effects of perceived subjective norms and prescriptive influence on financial decision-making **:
Wayne, (2022) addressed whether or not the respondent felt that society would approve or disapprove of certain actions. This pertains to an individual’s contemplation of the perceptions of their peers and individuals of significance toward their potential involvement in a particular behavior. Vyvyan et al., (2014) examined the impact of several factors on financial decision-making and effectiveness. The study’s findings indicated that learning behaviors exhibited within the familial context were perceived as influential in shaping individuals’ future financial behavior.
3) **The impact of perceived behavioral control on financial decision-making **:
Perceived behavioral control pertains to an individual’s subjective assessment of ease or difficulty associated with engaging in certain behaviors. The level of self-assurance that somebody possesses in their capability to perform a task substantially influences their subsequent actions. Self-efficacy is crucial in elucidating the connections among beliefs, attitudes, intentions, and behavior. Financial self-efficacy has been identified as a significant determinant of consumer financial decision-making power, as stated by Xiao et al. (2015). Similarly, it was also discovered that the perception of financial decision-making capabilities tends to rise with advancing age.

### 4.4. Theoretical contributions

In the face of these technological advancements and the fact that the aim of digital literacy is more or less aligned with the 2030 Agenda under the goal of women’s empowerment (and other sustainable development goals, or SDGs), the digital-financial space seems an appropriate context in which to apply the TPB, the TAM and other important models, and to test and refine them for broader applicability. This review of behaviors and technology adoption in this inter-integrated context should help us discern both the flexibility and the limitations of these models. Thinking about ‘perceived behavioral control’ in the context of digital financial literacy might also require a redefinition: perhaps when linked to digital finance, it would be appropriate to consider the existence of underlying social norms or even infrastructure constraints that might transform this construct considerably. In the TAM, we have ‘perceived usefulness’ related to the goal of digital finance, which refers less to the fact that the task is being completed than to more general life goals, such as empowerment and sustainability.

The integration of digital instruments into financial decision-making allows for the examination of the interplay between the TPB and TAM constructs. For instance, how does behavioral intention, as proposed by the theory of planned behavior (TPB), impact the usefulness of the technology acceptance model (TAM)? Findings could start to lay the framework for a unified model. Moreover, an analysis of cultural differences might help develop culturally adapted theoretical models, which are vital for global applicability. The environment may be different, hence generating the discovery of important new constructs or dimensions that are not incorporated in the existing theories. Community might be one of the influential factors that emerges in the acceptance of digital financial literacy. This review paper explores the interplay among digital and financial literacies, women’s emancipation, and sustainable development goals (SDGs), aiming to develop more holistic and situation-based frameworks consistent with current global challenges and expectations.

## 5. Conclusion

This article reviews the role of digital and financial literacy and women’s empowerment in their contribution to the Sustainable Development Goals (SDGs). Our findings show that there is a strong two-way causality between women’s digital and financial literacy and their empowerment. It also confirms that improving women’s literacy holistically not only empowers women but also facilitates the achievement of many SDGs. Digital and financial literacy are not only tools but also vehicles. They are bridges that take women across established social boundaries and connections towards other groups around the globe and more fully into the economy. We have seen that empowering women is pivotal to their countries’ sustainable growth, poverty alleviation and equal societies.

Large literacy gaps – most notably in Africa and Asia – involve challenges as well as opportunities. Smart investments in literacy in these areas promise to add a multiplicative effect and deliver payoffs in health, education and welfare. Many case studies and projects mentioned show that the route to women’s empowerment through digital and financial literacy, however difficult, is navigable and worthwhile. From grassroots initiatives to global campaigns, governments, nongovernmental organizations, and the corporate sector can work together to create an atmosphere favorable to learning and empowerment. There is a significant gender disparity in financial resilience, whereby men exhibit greater levels of financial resilience behaviors than women. A positive correlation exists between women who are employed and women who have attained higher levels of education and their ability to exhibit enhanced financial resilience ( Zeka & Alhassan, 2023).

## 6. Scope for future research

Digital and financial literacy and issues of gender – as they are associated with improved women’s empowerment and the fulfillment of sustainable development goals (SDGs) – is in itself a wide and fast-expanding field. The shifts and transformations we have observed over the past decade already highlight the vastness of potential vistas, the richest to be further explored by both academics and practitioners. A holistic, global framework, however, can only scratch the surface of what it takes to advance the field and create the best conditions for all women to become fully included in the gains of the digital and financial worlds. As much as the socioeconomic indicators aggregate on the surface, complexities inherent in regional scenarios and diversity require more granular analysis. It is justifiably helpful to deepen the insights from the variations of digital and gender societies in more specific contexts, such as the Southeast Asian landscape, where fast urbanization and cultural factors create an interesting dynamic, or in the Middle East, where sociopolitical factors strongly influence women’s trajectory in both digital and financial spheres. Even within regions, differences can be significant. In Africa, for instance, the gap between the pastoral settlements in the Sahel and the urban settings in South Africa can be radical, each presenting its own set of challenges and opportunities.

We also need to understand the growing digital divide: while urban areas – as well as the industrialized parts of the world – are moving towards a hyper-connected future, many people, especially women in rural areas, are left behind, without even basic digital infrastructure. Solutions to close the gap, from low-tech digital interventions to community-level initiatives, need to be researched. The role of the private sector, particularly large technology, and financial companies is also worth investigating. Linking other identity components, such as ethnicity, socioeconomic status or age, to gender offers another avenue to a richer, more nuanced understanding.

## 7. Policy implications

The findings of the digital and financial literacy paradigm of women’s empowerment and the achievement of the SDGs have implications for all strata of people, which creates disruptive changes at the individual and societal levels. The implications at the policy frontier is far-reaching. Policymakers have a data-driven paradigm to guide them to develop new evidence-based approaches to advancing gender-equitable empowerment in new spaces. The findings highlight the need to teach digital and financial literacy in existing academic curriculums and for education institutions to evolve into knowledge spaces that foster not just the delivery of the requisite skills to navigate contemporary society but also their development. The implications of the study of digital and financial literacies, women’s empowerment, and their SDGs transcend academic endeavors and are relevant in the policymaking, NGO efforts, technology company mission, and global development strategy space. These implications are particularly pertinent to the synergies between digital and financial literacies, women’s empowerment and the SDGs.

## Data Availability

Data sharing is not applicable—no new data are generated, or the article describes entirely theoretical research.
