# Supplementary material for: Digital and Financial Literacy for Uplifting Women and Achieving Sustainable Development Goals
Source: F1000Res. 2026 Mar 13;13:1364. Originally published 2024 Nov 14. [Version 2] doi: 10.12688/f1000research.156744.2 (PMC13033132; doi:10.12688/f1000research.156744.2)
Supplement: Peer review history

# Reviewer report: Reviewer response for version 2

**DOI:** 10.5256/f1000research.180403.r468063

**Published:** 2026-03-28

**Authors:** Ekta Sharma

**Affiliations:** 1 Ahmedabad University, Ahmedabad, Gujarat, India

The study addresses a critical intersection of **S**DG 5 (Gender Equality) and SDG 8 (Decent Work and Economic Growth), but there are specific areas where the "Systematic" nature of the review and the "Theoretical Depth" can be strengthened.The revised version of your systematic review shows significant progress, particularly in its methodological transparency (PRISMA) and currency of citations.

 To elevate this work further, I recommend that you move from a chronological or study-by-study summary toward a **Thematic Synthesis**. Categorizing findings into pillars—such as 'The Access-Usage Gap' or 'Psychological Barriers to Digital Resilience'—would provide more utility to future researchers.

 Furthermore, I suggest you propose a Conceptual Model in your discussion that illustrates the pathway from digital literacy to financial resilience, specifically moderated by the unique constraints faced by working women (e.g., the 'double-burden' or household hierarchy)."

 Also please check for some minor issues like in abstract you say 'improvement of the SDG', technically this makes no sense as you are discussing achievement of the SDG instead.

Is the review written in accessible language?

Yes

Are all factual statements correct and adequately supported by citations?

Yes

Are the conclusions drawn appropriate in the context of the current research literature?

Yes

Is the topic of the review discussed comprehensively in the context of the current literature?

Yes

Reviewer Expertise:

Sustainability, ethics , innovation & creativity and behaviour

I confirm that I have read this submission and believe that I have an appropriate level of expertise to confirm that it is of an acceptable scientific standard, however I have significant reservations, as outlined above.

# Reviewer report: Reviewer response for version 2

**DOI:** 10.5256/f1000research.180403.r468059

**Published:** 2026-03-28

**Authors:** Vikas Garg

**Affiliations:** 1 Symbiosis International (Deemed University), Pune, Maharashtra, India

The article titled *"Digital and Financial Literacy for Uplifting Women and Achieving Sustainable Development Goals"* presents a systematic literature review (SLR) using the PRISMA approach to examine how digital literacy and financial literacy contribute to women's empowerment and the achievement of Sustainable Development Goals (SDGs).

 The authors reviewed 1,045 initial articles, narrowing them down to 85 studies, primarily focused on India and other emerging economies. The study identifies that:

- Digital literacy enhances access to information, networks, and entrepreneurial opportunities.
- Financial literacy improves decision-making, financial independence, and economic participation.
- The integration of both literacies acts as a catalyst for poverty reduction, gender equality, and sustainable development.
- Key barriers include lack of infrastructure, education gaps, socio-cultural constraints, and gender inequality.

The paper also attempts to link findings with theoretical frameworks such as Theory of Planned Behavior (TPB) and Technology Acceptance Model (TAM).

Is the review written in accessible language?

Yes

Are all factual statements correct and adequately supported by citations?

Yes

Are the conclusions drawn appropriate in the context of the current research literature?

Yes

Is the topic of the review discussed comprehensively in the context of the current literature?

Yes

Reviewer Expertise:

Digital literacy, financial literacy

I confirm that I have read this submission and believe that I have an appropriate level of expertise to confirm that it is of an acceptable scientific standard.

# Reviewer report: Reviewer response for version 2

**DOI:** 10.5256/f1000research.180403.r468065

**Published:** 2026-03-28

**Authors:** Nripendra P Rana

**Affiliations:** 1 Queen's University Belfast, Belfast, Northern Ireland, UK

The paper is well written and I will accept it in its current form. The literature review is very comprehensive. The introduction is well motivated. Findings are well articulated. Theoretical contributions are clearly highlighted. I would only suggest that the authors should write policy implications in parallel to theoretical contributions. The authors should also include some limitations of this research.

Is the review written in accessible language?

Yes

Are all factual statements correct and adequately supported by citations?

Yes

Are the conclusions drawn appropriate in the context of the current research literature?

Yes

Is the topic of the review discussed comprehensively in the context of the current literature?

Yes

Reviewer Expertise:

IS/IT Adoption and Diffusion

I confirm that I have read this submission and believe that I have an appropriate level of expertise to confirm that it is of an acceptable scientific standard.

# Reviewer report: Reviewer response for version 2

**DOI:** 10.5256/f1000research.180403.r467447

**Published:** 2026-03-23

**Authors:** Nurul Hidayana Mohd Noor

**Affiliations:** 1 Faculty of Administrative Science and Policy Studies, Universiti Teknologi MARA, Seremban, Negeri Sembilan, Malaysia

The authors have made the necessary amendments as requested.

Is the review written in accessible language?

Partly

Are all factual statements correct and adequately supported by citations?

Partly

Are the conclusions drawn appropriate in the context of the current research literature?

Partly

Is the topic of the review discussed comprehensively in the context of the current literature?

Partly

Reviewer Expertise:

Women and gender studies, entrepreneurship, youth studies, and organizational management.

I confirm that I have read this submission and believe that I have an appropriate level of expertise to confirm that it is of an acceptable scientific standard.

# Reviewer report: Reviewer response for version 1

**DOI:** 10.5256/f1000research.172103.r348865

**Published:** 2024-12-17

**Authors:** Maria Alzira Pimenta Dinis

**Affiliations:** 1 Universidade Fernando Pessoa, Porto, Portugal

It is unclear the region addressed in the title or if global scope

 The abstract would need to clarify the specific findings as further practical implications

 The Introduction needs to cite more recent references as connecting paragraphs. This is applied to the entire piece of text

 The literature review reveals some expressive similarity, which is also present I the methods section and the discussion section (there seems to be no results section…)

 All the content mentioned here needs references, as it resulted from the literature review:

 “4.1 Synergy between digital, financial literacy, women’s empowerment and SDGs”

 It is not clear to me why this is the point two of the “discussion”: “4.2 Underpinning theories”

 This part needs references again and further connection

 “4.4. Theoretical contributions”

 The conclusions section could address context and methods, findings and implications, limitations and future prospects, all here.

 The entire articles needs to address full cohesion and streamlining

 Authors needs to highlight novelty and relevance, compared to existing studies.

 All older references should be revised

 As presented, the text is just too descriptive, missing the necessary tables and graphics or schemes

Is the review written in accessible language?

Partly

Are all factual statements correct and adequately supported by citations?

Partly

Are the conclusions drawn appropriate in the context of the current research literature?

No

Is the topic of the review discussed comprehensively in the context of the current literature?

No

Reviewer Expertise:

Sustainability

I confirm that I have read this submission and believe that I have an appropriate level of expertise to state that I do not consider it to be of an acceptable scientific standard, for reasons outlined above.

# Reviewer report: Reviewer response for version 1

**DOI:** 10.5256/f1000research.172103.r341083

**Published:** 2024-12-02

**Authors:** Nurul Hidayana Mohd Noor

**Affiliations:** 1 Faculty of Administrative Science and Policy Studies, Universiti Teknologi MARA, Seremban, Negeri Sembilan, Malaysia

It is an exciting topic, yet several suggestions exist for improving the current paper. The recommendations are as follows:

1. Most of the citations in the introduction part are obsolete. Please replace them with the recent ones.
2. More sentences should be added to the introduction to highlight the issues faced by women (lack of financial literacy and digital financial literacy). Please provide evidence and statistics.
3. In the LR section, some sections are irrelevant (e.g., financial resilience and women empowerment). Perhaps you can combine these two sections into one section, such as The Challenges faced by Women in Achieving Financial Well-Being. Thus, under this one section, the authors can discuss the financial well-being concept, financial constraints, and strategies to empower women to achieve financial well-being. Then, follow with the financial literacy section and digital financial literacy section. Please make sure all citations are at the latest (2019-2024). And please diversify your citations.
4. The SLR technique is not straightforward in the methodology section because the authors do not determine the method used. For example, ROSES or PRISMA.
5. It would be better if the researchers could provide diagrams for conducting SLR. For example, the PRISMA flow diagram (please check on it). The inclusion and exclusion criteria for articles need to be clearly stated.
6. Discussion- rename it into findings and discussion
7. The most critical flaw in this article is your findings/discussion part, where the researchers are encouraged to organize it into research objectives. Because the readers are not clear from the introduction section.  For example, in the introduction section, the authors need to list out the sub-objectives of their paper. For example:

1. To identify the impact of financial literacy
2. To assess the importance of digital financial literacy
3. For instance, the current trends of research in financial and financial digital literacy (i.e., the theory underpinning and methodological aspects)
4. Xxxx

Thus, organizing your findings and discussion will be more apparent because you can discuss your SLR findings based on the objective. The current content is confusing and not clear. After you have presented your findings. You can consult the theoretical, policy, or practical contributions and close the manuscript with the conclusion section.

Is the review written in accessible language?

Partly

Are all factual statements correct and adequately supported by citations?

Partly

Are the conclusions drawn appropriate in the context of the current research literature?

Partly

Is the topic of the review discussed comprehensively in the context of the current literature?

Partly

Reviewer Expertise:

Women and gender studies, entrepreneurship, youth studies, and organizational management.

I confirm that I have read this submission and believe that I have an appropriate level of expertise to confirm that it is of an acceptable scientific standard, however I have significant reservations, as outlined above.

# Response

**Published:** 2024-12-14

**Authors:** Vinay Kandpal

The recommendations are as follows:

1. Most of the citations in the introduction part are obsolete. Please replace them with the recent ones.- **Literature updated in Introduction and LR**
2. More sentences should be added to the introduction to highlight the issues faced by women (lack of financial literacy and digital financial literacy). Please provide evidence and statistics. **Literature and supporting statistics updated in Introduction and LR**
3. In the LR section, some sections are irrelevant (e.g., financial resilience and women empowerment). Perhaps you can combine these two sections into one section, such as The Challenges faced by Women in Achieving Financial Well-Being. Thus, under this one section, the authors can discuss the financial well-being concept, financial constraints, and strategies to empower women to achieve financial well-being. Then, follow with the financial literacy section and digital financial literacy section. Please make sure all citations are at the latest (2019-2024). And please diversify your citations. **Diversified citations included. **The suggested two sections clubbed into one section
4. The SLR technique is not straightforward in the methodology section because the authors do not determine the method used. For example, ROSES or PRISMA. **PRISMA included in Research Methods**
5. It would be better if the researchers could provide diagrams for conducting SLR. For example, the PRISMA flow diagram (please check on it). The inclusion and exclusion criteria for articles need to be clearly stated. **PRISMA included in Research Methods** stating the inclusion and exclusion.
6. Discussion- rename it into findings and discussion: **Suggested Changes made in the revised paper.**
7. The most critical flaw in this article is your findings/discussion part, where the researchers are encouraged to organize it into research objectives. Because the readers are not clear from the introduction section.  For example, in the introduction section, the authors need to list out the sub-objectives of their paper. For example:

1. To identify the impact of financial literacy
2. To assess the importance of digital financial literacy
3. For instance, the current trends of research in financial and financial digital literacy (i.e., the theory underpinning and methodological aspects

Thus, organizing your findings and discussion will be more apparent because you can discuss your SLR findings based on the objective. The current content is confusing and not clear. After you have presented your findings. You can consult the theoretical, policy, or practical contributions and close the manuscript with the conclusion section.

**Suggested Changes incorporated in revised Version of paper.**
